# Analysis of Wilson disease mutations revealed that interactions between different ATP7B mutants modify their properties

**DOI:** 10.1038/s41598-020-70366-7

**Published:** 2020-08-10

**Authors:** Shubhrajit Roy, Courtney J. McCann, Martina Ralle, Kunal Ray, Jharna Ray, Svetlana Lutsenko, Samuel Jayakanthan

**Affiliations:** 1 Department of Physiology, Johns Hopkins Medical Institute, Baltimore, MD USA; 2 Oregon Health and Science University, Portland, OR USA; 3 ATGC Diagnostics Private Ltd, Kolkata, India; 4 S. N. Pradhan Centre for Neurosciences, University of Calcutta, Kolkata, India

**Keywords:** Cell biology, Genetics, Molecular biology

## Abstract

Wilson disease (WD) is an autosomal-recessive disorder caused by mutations in the copper (Cu)-transporter *ATP7B*. Thus far, studies of WD mutations have been limited to analysis of *ATP7B* mutants in the homozygous states. However, the majority of WD patients are compound-heterozygous, and how different mutations on two alleles impact ATP7B properties is unclear. We characterized five mutations identified in Indian WD patients, first by expressing each alone and then by co-expressing two mutants with dissimilar properties. Mutations located in the regulatory domains of ATP7B—A595T, S1362A, and S1426I—do not affect ATP7B targeting to the trans-Golgi network (TGN) but reduce its Cu-transport activity. The S1362A mutation also inhibits Cu-dependent trafficking from the TGN. The G1061E and G1101R mutations, which are located within the ATP-binding domain, cause ATP7B retention in the endoplasmic reticulum, inhibit Cu-transport, and lower ATP7B protein abundance. Co-expression of the A595T and G1061E mutations, which mimics the compound-heterozygous state of some WD patients, revealed an interaction between these mutants that altered their intracellular localization and trafficking under both low and high Cu conditions. These findings highlight the need to study WD variants in both the homozygous and compound-heterozygous states to better understand the genotype–phenotype correlations and incomplete penetrance observed in WD.

## Introduction

Copper (Cu) is a redox-active metal that is indispensable for human growth and development. Cu is required for the activity of several enzymes, including cytochrome c oxidase, Cu/Zn-dependent superoxide dismutases, tyrosinase, dopamine-β-hydroxylase, ceruloplasmin, and peptidyl-α-monooxygenase^1^. In order to ensure an adequate supply of Cu without toxic accumulation, Cu homeostatic mechanisms are tightly regulated. Either Cu deficiency or overabundance is associated with pathologic changes and disease, such as, Menkes disease or Wilson disease (WD), respectively^2,3^. WD is a genetic disorder caused by mutations in the Cu-transporter *ATP7B* that causes abnormal deposition of Cu in the liver and the brain^4^. WD has a spectrum of hepatic and neurological manifestations, along with a broad range of disease onsets^5^. The mechanisms behind this variability remain poorly understood. Studies of WD patients identified over 600 *ATP7B* mutations^6^ and revealed the diverse effects of mutations on the functional and cellular properties of the ATP7B protein. Efforts to link different phenotypic presentations of WD to specific *ATP7B* mutations have not produced strong correlations and have sometimes led to conflicting results^7^. High prevalence of compound-heterozygous mutations further complicates the task of genotype–phenotype correlation^8^.

ATP7B is a 164 kDa, multi-domain protein that transfers Cu across the cell membrane using energy generated by ATP hydrolysis^9–11^. ATP7B resides in the *trans*-Golgi network (TGN), where it supplies Cu for the functional maturation of the Cu-dependent enzyme ceruloplasmin. In response to Cu elevation, ATP7B undergoes kinase-mediated phosphorylation and moves out of the TGN to vesicles, where it sequesters excess Cu for subsequent export across the apical membrane^12^. The complexes, multi-domain structure of ATP7B, combined with a limited understanding of the mechanisms underlying ATP7B targeting and trafficking, have led to difficulties in determining the precise consequences of pathogenic mutations. In addition, ATP7B forms stable dimers both in cells and in-vitro, and very little is yet known about the significance of its oligomerization^18^. Dimerization with wild-type (WT) ATP7B was shown to affect the localization of ATP7B variants with mutated kinase-mediated phosphorylation sites^13^https://www.ncbi.nlm.nih.gov/pmc/articles/PMC3476272/, although the impact of dimerization on the properties of WD mutants has not been investigated. It remains unclear whether different ATP7B mutants expressed in the same cells act independently or influence each other’s properties.

**Figure 1:**
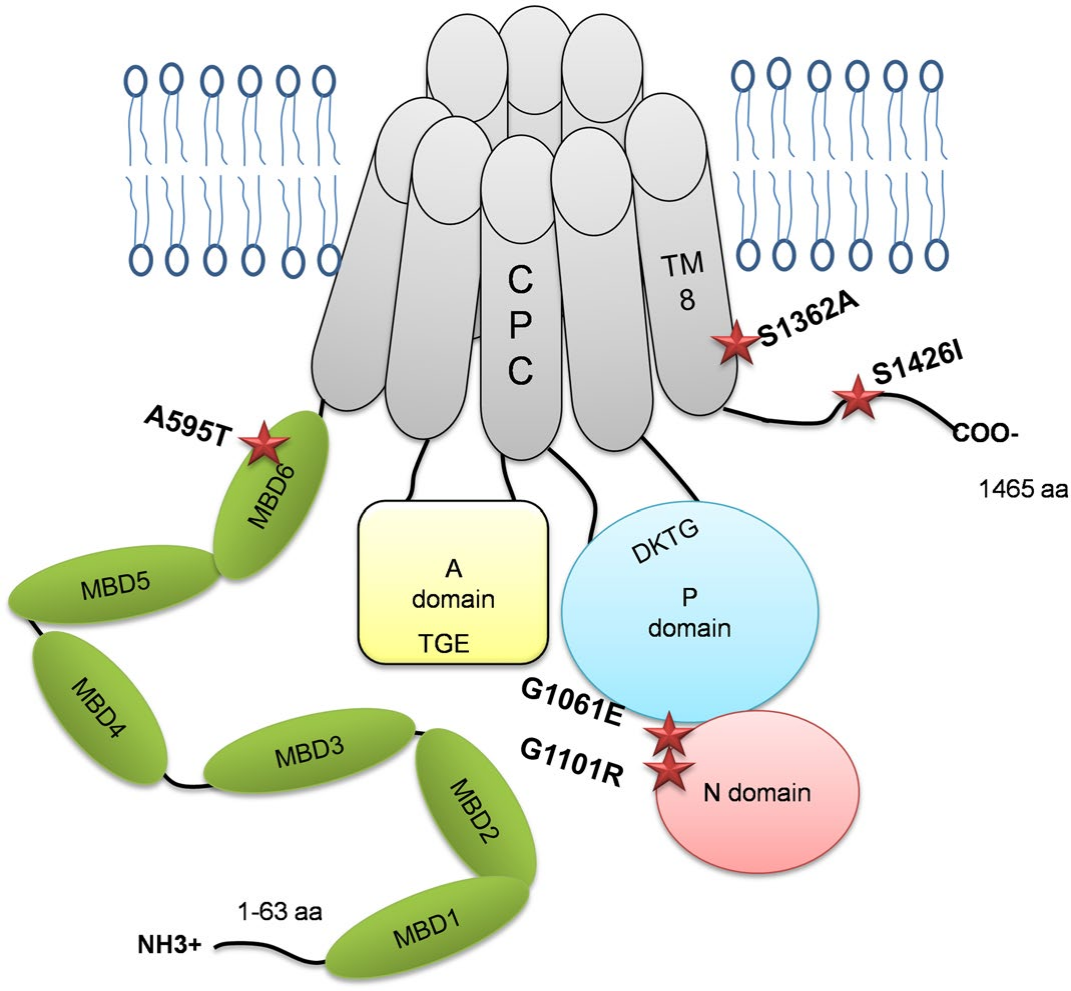
Locations of selected WD mutations in the ATP7B protein. Diagrammatic representation of the ATP7B protein structure and the positions of the ATP7B mutations (A595T, G1061E, G1101R, S1362A, and S1426I) in different domains.

Extensive genetic screening of *ATP7B *among Indian WD patients identified several novel and prevalent missense mutations present in both homozygous and compound-heterozygous states^7^. In this study, we characterized mutations found in different domains of ATP7B (Fig. 1). The previously reported mutations G1061E and G1101R are located within the ATP-binding domain and were detected in both homozygous and heterozygous states. Three novel mutations—A595T, S1362A and S1426I—are located in the N-terminal metal binding domain 6 (MBD 6), trans-membrane domain 8 (TM 8), and the C-terminal domain, respectively (Fig. 1). The S1362A mutation was found in the homozygous state, whereas A595T and S1426I were found in compound-heterozygous WD patients.

We hypothesized that WD-causing mutations located in different domains of ATP7B would have different impacts on its Cu-transport activity or trafficking behavior. We tested this hypothesis by characterizing the properties of the individual *ATP7B* mutants in isolation, i.e., as they are found in the homozygous state. We then modeled the compound-heterozygous state found in WD patients by co-expressing the A595T and G1061E mutants and characterizing their cellular behavior under low and high Cu conditions.

## Materials and methods

### Plasmids and site-directed mutagenesis

The plasmid pSJ101, encoding full-length *ATP7B* with an N-terminal 6x-His-GFP-TEV tag (6X-His-GFP-tev-ATP7B), and another plasmid encoding full-length ATP7B with an N-terminal Flag-tag (pcDNA5 FRT/TO plasmid; Invitrogen) were used as templates to generate the missense mutations. The *ATP7B* mutations were introduced using the Quick-Change site-directed mutagenesis kit (Stratagene, La Jolla, CA) and appropriate primers (Table S1). Correct sequences and the presence of the mutations were verified by sequencing the entire cDNA region. The sequencing was performed by the Johns Hopkins School of Medicine Synthesis and Sequencing Facility.

### Cell culture and transient transfection

HEK293A cells were maintained in Dulbecco’s Modified Eagle’s Medium (DMEM; Corning Cellgro, Fisher Scientific, USA) supplemented with 1% penicillin/streptomycin and 10% fetal bovine serum (FBS; vol/vol). Menkes disease fibroblast (YST) cells were maintained in DMEM supplemented with 1% penicillin/streptomycin, 0.5 µg/mL Puromycin (Invitrogen, Carlsbad, CA, USA), Primocin (Invitrogen, Carlsbad, CA, USA), and 10% FBS (vol/vol) (Corning Cellgro, USA). Cell cultures were maintained at 37 °C in a humidified incubator (5% CO2). The cells were transfected in 6-well plates for 12–18 h using Lipofectamine LTX-PLUS reagent (Invitrogen, Carlsbad, CA, USA) with 1 or 2 µg of plasmid DNA and Opti-MEM reduced serum medium (Gibco; life-technologies, USA) following the manufacturer’s protocol.

### Tyrosinase activation assay for determining Cu-transport activity

YST cells were seeded onto sterilized, 22 × 22 mm^2^ glass coverslips at a density of 2.5 × 10^5^ cells per well. The cells were transfected with either 1 µg of plasmid expressing tyrosinase (human tyrosinase protein in pcDNA 3.1 A ()myc/His) alone or with 1 μg each of pTYR and the 6X HisGFP-tev-ATP7B plasmids (WT, S1362A, A595T, S1426I, G1101R, or G1061E) as described above. After 16–18 h of transfection, the cells were washed twice in 0.1 M sodium phosphate buffer (pH 6.8) and fixed for 30 s in ice-cold acetone-methanol mix (1:1). The cells were then incubated for 3 h at 37 °C in 0.1 M sodium phosphate buffer (pH 6.8) containing 0.4 mg/mL levo-3,4-dihydroxy-L-phenylalanine (L-DOPA)^14^. Coverslips were mounted on slides using Fluoromount-G (Electron Microscope Sciences, USA), and the formation of black eumelanin pigment was detected by phase contrast microscopy. A previously characterized catalytically inactive mutant D1027A^15^ has been used as a negative control in the tyrosinase activity assay. Pigment intensity and area were quantified using Image-J software^16^, and total signal intensity was calculated using the equation

$$total \,signal\, intensity = pigment\, intensity \times pigment \,area.$$

The expression of ATP7B in YST cells were estimated using confocal microscopy by measuring GFP-fluorescence intensity normalized to its area.

### SDS PAGE and Western blot

YST cells were transfected with 2 µg of the 6X His-GFP-TEV-ATP7B plasmids as described above. Following protein expression, cells were lysed for 30 min on ice using 250 µL of RIPA lysis buffer (0.05 M Tris–HCl, pH 7.0; 0.15 M NaCl; 0.25% deoxycholic acid; 1% NP-40; and 1 mM EDTA) supplemented with 1 Complete EDTA-free protease inhibitor tablet (Roche) and 100 µM of AEBSF (Sigma-Aldrich, USA). The cells were centrifuged at 3000×*g* for 15 min to remove cell debris. The supernatant was collected and used as whole cell lysate for further studies. Protein concentration was determined by BCA assay (Pierce).

Approximately 20 µg of total protein was separated by SDS-PAGE on 10% Laemmli gels (BioRad, USA). The proteins were then transferred to PVDF membranes (Millipore, USA) using 10 mM CAPS buffer, pH 11. Membranes were blocked for 1 h at room temperature (RT) in 5% milk diluted in phosphate-buffered saline (PBS). The membranes were incubated in rabbit anti-ATP7B (1:500; Abcam) or mouse anti-β-actin(1:2000, Abcam) primary antibodies for 1 h at RT and then goat anti-rabbit IgG-HRP (1:10,000; Santa Cruz) or sheep anti-mouse IgG-HRP (1:10,000; GE Healthcare) secondary antibodies for 1 h at RT. The membranes were treated with horseradish peroxidase (HRP) substrate for enhanced chemiluminescence (ECL; Pierce), and the protein bands were visualized with an Amersham Imager 600 system (GE healthcare). The fluorescent band intensities of the proteins were quantified with Image-J software (NIH). β-actin was used as a loading control.

### Confocal fluorescence microscopy and trafficking studies:

HEK293 cells were seeded onto flame sterilized, 22 × 22 mm^2^ glass coverslips at a density of 2 × 10^5^ cells in 6-well plates. The cells were grown to 70–80% confluency and then transiently transfected with 1 µg of DNA (WT or mutant) as described above. After 12 h of transfection, the cells were incubated in media containing 9 µg/mL cycloheximide and either 25 µM tetrathiomolybdate (TTM; low Cu) or media containing 100 µM CuCl_2_ (high Cu) for 3 h. The cells were then fixed with 4% paraformaldehyde (PFA) diluted in PBS for 15 min at RT, permeabilized with 0.5% Triton X-100 (Sigma) for 15 min at RT, and blocked in 5% BSA in PBS for 45 min at RT. Cells were incubated with sheep anti-TGN46 (1:300 dilution; Novus Biotech) or goat anti-calnexin (1:300; Sigma) primary antibodies for 1 h at RT and then incubated with donkey anti-sheep AlexaFluor-555 (Invitrogen) or donkey anti-goat AlexaFluor-633 (Invitrogen) secondary antibodies for 1 h at RT. Coverslips were mounted using 3:1 Fluoromount-G + DAPI (Electron Microscopy Science). Stained cells were imaged using a Zeiss LSM800 microscope with a Plan-Apochromat 63x/1.4 NA oil lens, and the images were then processed using Image-J.

### Inductively coupled plasma mass-spectrometry (ICP-MS) for Cu measurements

YST cells were transfected with 2 µg of the ATP7B plasmids as described above. After 16 h of transfection, the cells were treated with 100 μM CuCl_2_ for 24 h and then collected by centrifugation at 10,000 rpm for 10 min. Approximately 10^6^ cells were collected from each well and then washed once with ultrapure water. The cell pellets were resuspended in 100 µL of concentrated nitric acid (trace element grade, Fisher Scientific) and heated at 90 °C for 2 h. The samples were cooled to RT and then diluted in 1.4 mL of 1% nitric acid. The volume of the sample was determined before ICP-MS measurements were taken. The concentration of Cu was normalized to cell count.

### Statistical analysis

All data were analyzed using Graphpad Prism 8 software unless otherwise noted. All values are reported as means ± standard deviation (SD), as indicated, from at least 3 independent tests. Statistical significance was determined using either unpaired Student’s *t* test or one-way ANOVA with Dunnett’s post-hoc test against the control or WT, as indicated.*p < 0.05; **p < 0.01; ***p < 0.001, and ****p < 0.0001.

## Results

Defining the cellular properties of WD-causing mutations helps to establish genotype–phenotype correlations and facilitates the development of mutation-specific disease monitoring and therapeutic approaches. In this study, we have characterized the expression, localization, activity, and trafficking patterns of several WD mutations commonly found in the Indian population. The selected mutations are located in domains essential for the ATP-binding and Cu-transport activities of ATP7B, as well as in the regulatory N-terminal and C-terminal domains. We hypothesized that the consequences of the mutations will differ between those in the functional domains and those in the regulatory domains.

### A595T ATP7B mutant has reduced Cu-transport activity but normal localization and trafficking behavior

**Figure 2:**
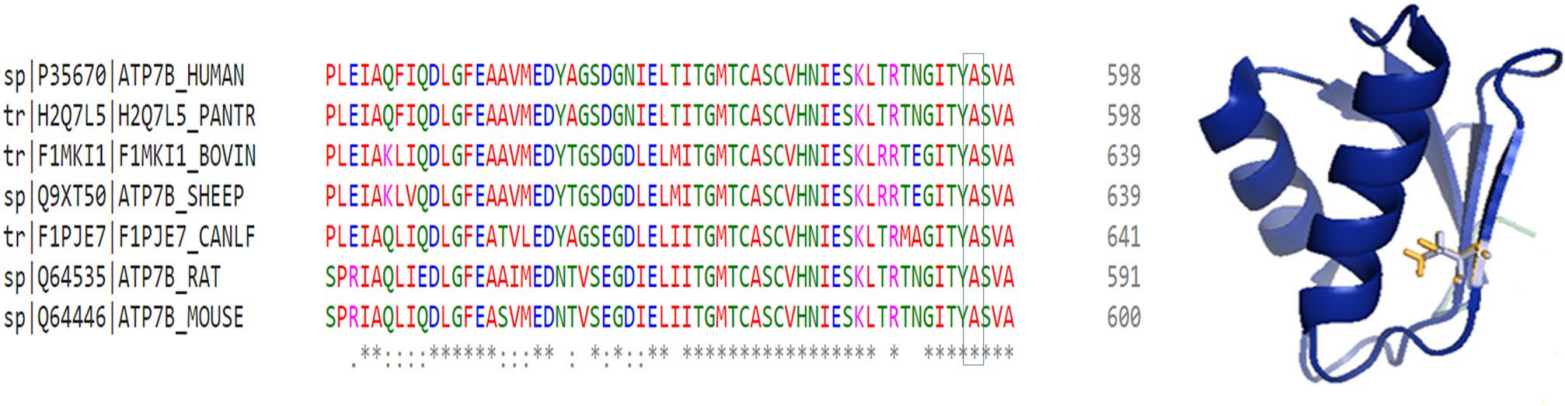
Conservation and location of Ala595 in MBD6. (**A**) Multiple sequence alignment using Clustal-omega (EMBL-EBI) of the ATP7B protein showing the conservation status of the Ala595 residue among several mammalian species (Human, chimpanzee, cow, sheep, dog, rat,andmouse). (**B**) The structure of MBD6 (PDB accession 2EW9) with the Ala substituted with Thr using the mutagenesis function in PyMol. The difference in length of the Ala and Thr side chains are indicated in yellow.

The A595T mutation was originally reported in a male WD patient in the compound-heterozygous state with the G1061E mutation on the other allele (Table S2)^7^. The Ala residue in this position is evolutionarily conserved, as evidenced by the alignment of ATP7B sequences from multiple species (Fig. 2a). A595 is located in the N-terminal domain on the β strand of MBD6, with its side chain projecting into the protein core (Fig. 2b). Substitution of Ala (Van der Waals volume A^3^: 67 and accessible surface area A^2^: 67) with Thr (Van der Waals volume A^3^: 93 and accessible surface area A^2^: 102) (Table S3) increases the bulk of the side chain (Fig. 2b, extra volume indicated in yellow) and replaces the uncharged hydrophobic Ala with a partially charged Thr in the vicinity of Leu and Glu. Thus, the A595T change, although relatively small, could be deleterious.

**Figure 3:**
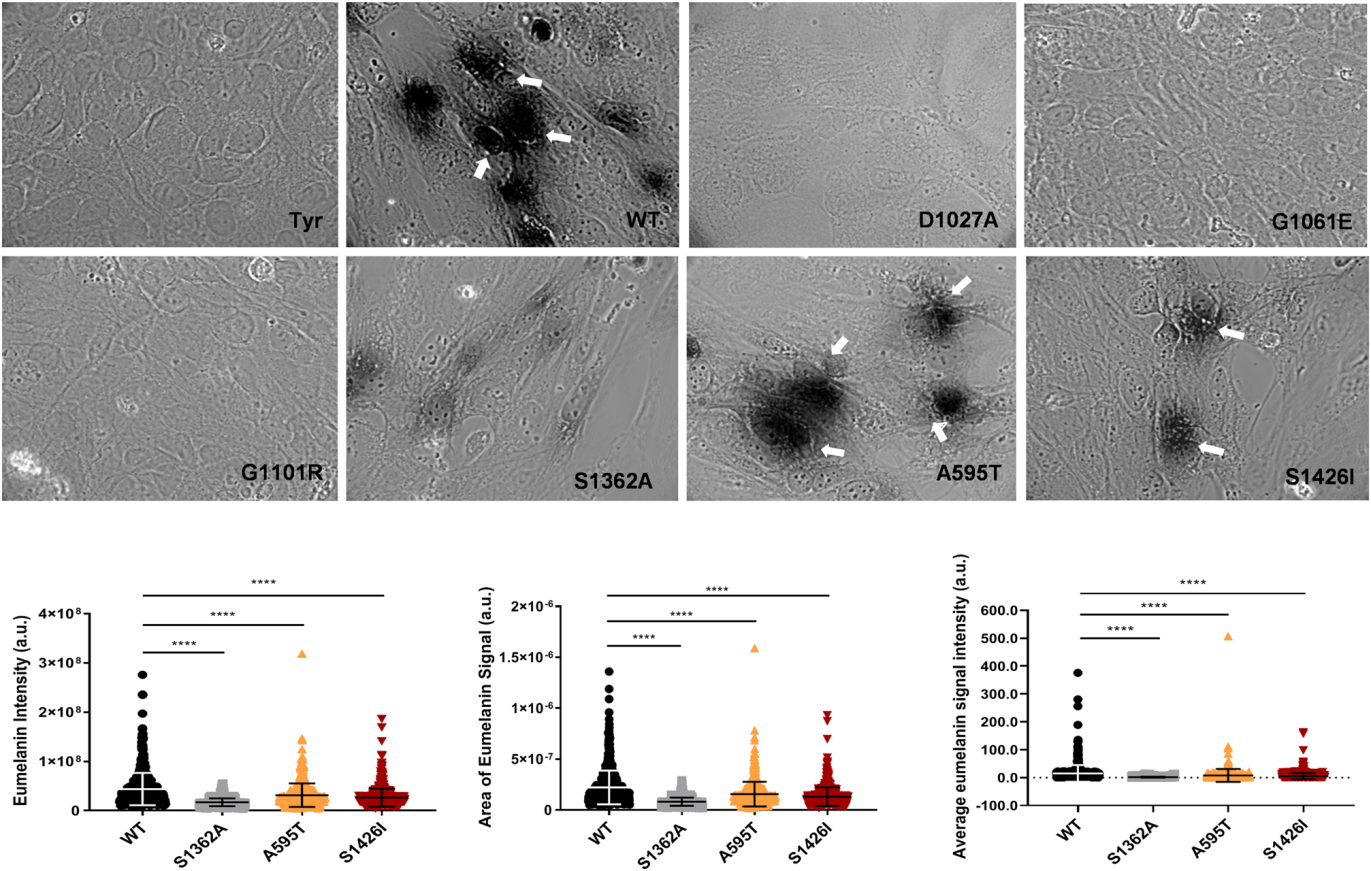
Cu-transport activity of the ATP7B mutants. (**A**) YST Cells were transfected with the tyrosinase-encoding plasmid alone (Tyr, negative control) or together with wild-type (WT) ATP7B, the catalytically inactive D1027A mutant (negative control), or the ATP7B mutants found in WD patients. Cu-transport activity was evaluated by measuring eumelanin pigment formation (indicated with white arrows).The area (**B**) and intensities (**C**) of the pigment were quantified. (**D**) Total eumelanin pigment signal (area × intensity) in cells with the WD mutations were quantified and compared to the WT values. All values are reported as means ± SD (Standard deviation). Significance was determined by one-way ANOVA with Dunnet’s multiple comparison test, ****p < 0.0001, *n* = 3.

**Figure 4:**
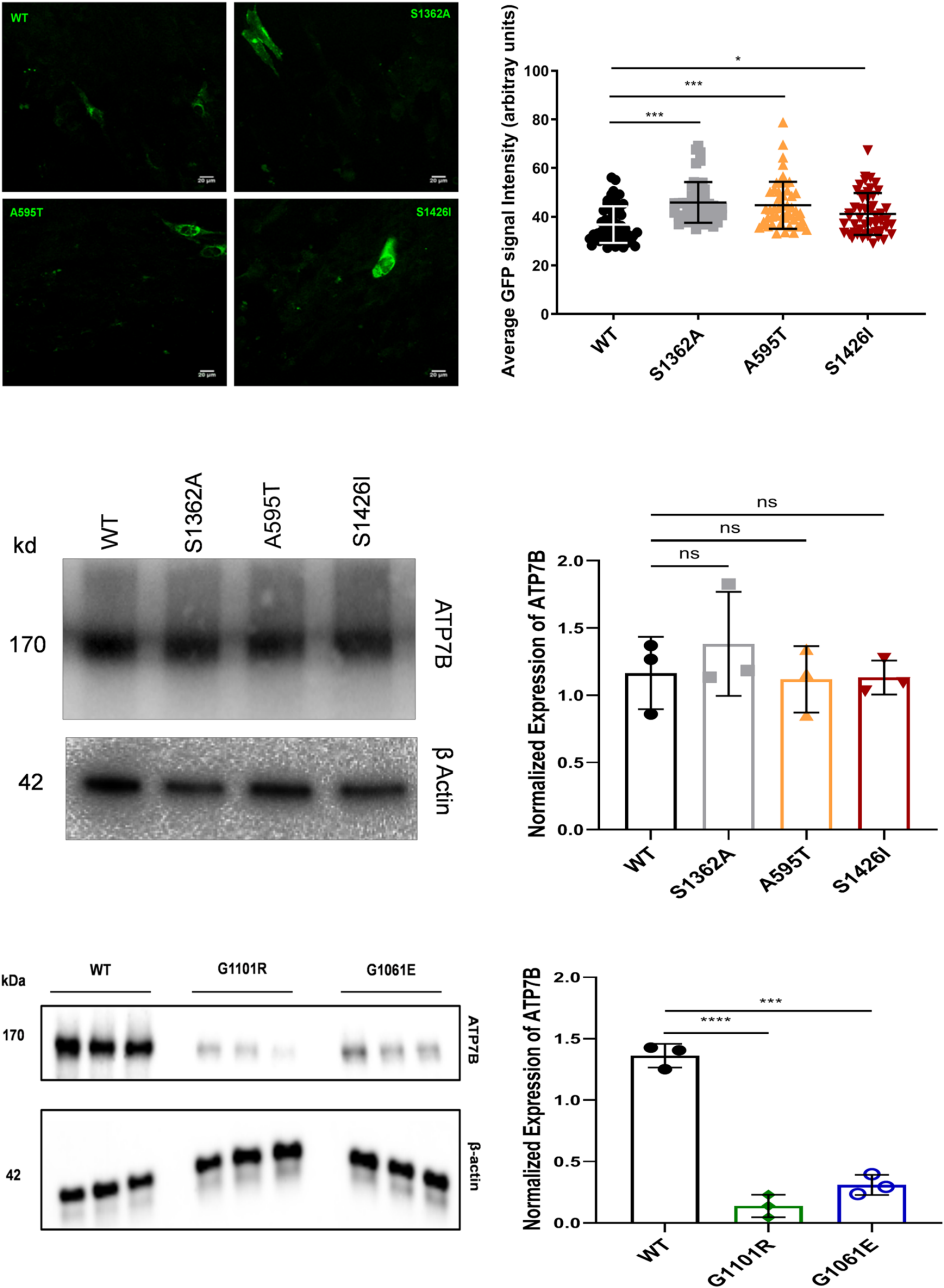
Expression of WT and mutant ATP7B proteins in YST cells. (**A**) The expression of GFP-tagged WT ATP7B and the Ala595Thr (A595T), Ser1362Ala (S1363A), and Ser1426Ile (S1426I) ATP7B mutants in YST cells visualized using GFP fluorescence. (**B**) The intensity of GFP fluorescence was normalized to the area. All values are reported as means ± SD. Significance was determined by one-way ANOVA with Dunnet’s multiple comparison test, *p < 0.05 and ***p < 0.001. (**C**) Representative Western blot and (**D**) densitometric analysis of the WT, S1362A, A595T, and S1426I protein abundances in YST cells. The band intensities were normalized to β-actin. All values are reported as means ± SD. Significance was determined by unpaired Student’s *t* test, *n* = 3. (**E**) Representative Western blot and (**F**) densitometric analysis of WT ATP7B and the Gly1101Arg (G1101R), and Gly1061Glu (G1061E) ATP7B mutant protein abundances in YST cells. The band intensities were normalized to β-actin. All values are reported as means ± SD. Significance was determined by one-way ANOVA with Dunnet’s multiple comparison test, ***p < 0.001; ****p < 0.0001, *n* = 3.

To directly test this prediction, we characterized the Cu-transport activity of the A595T variant. Analysis of Cu-transport by this and other mutants was monitored in Menkes disease fibroblast (YST) cells, which lack active ATP7A and ATP7B. As a result, the transfer of Cu to the secretory pathway (TGN and vesicles) is impaired, and cells produce inactive tyrosinase, a pigment-generating Cu-dependent enzyme. Expression of WT ATP7B restores Cu delivery to tyrosinase and activates the enzyme, which can be monitored by the appearance of dark eumelanin pigment (Fig. 3). Expression of the A595T mutant resulted in pigment formation, indicating that this mutant has Cu-transport activity. However, the total intensity of the signal was 52.4% lower than that of WT ATP7B (15.11159 ± 29.8 vs. 7.91 ± 23.04, respectively; Fig. 3). Quantitation of GFP signal in cells and analysis of protein abundance indicate that the lower activity of the mutant is not the result of lower protein expression (Fig. 4).

**Figure 5:**
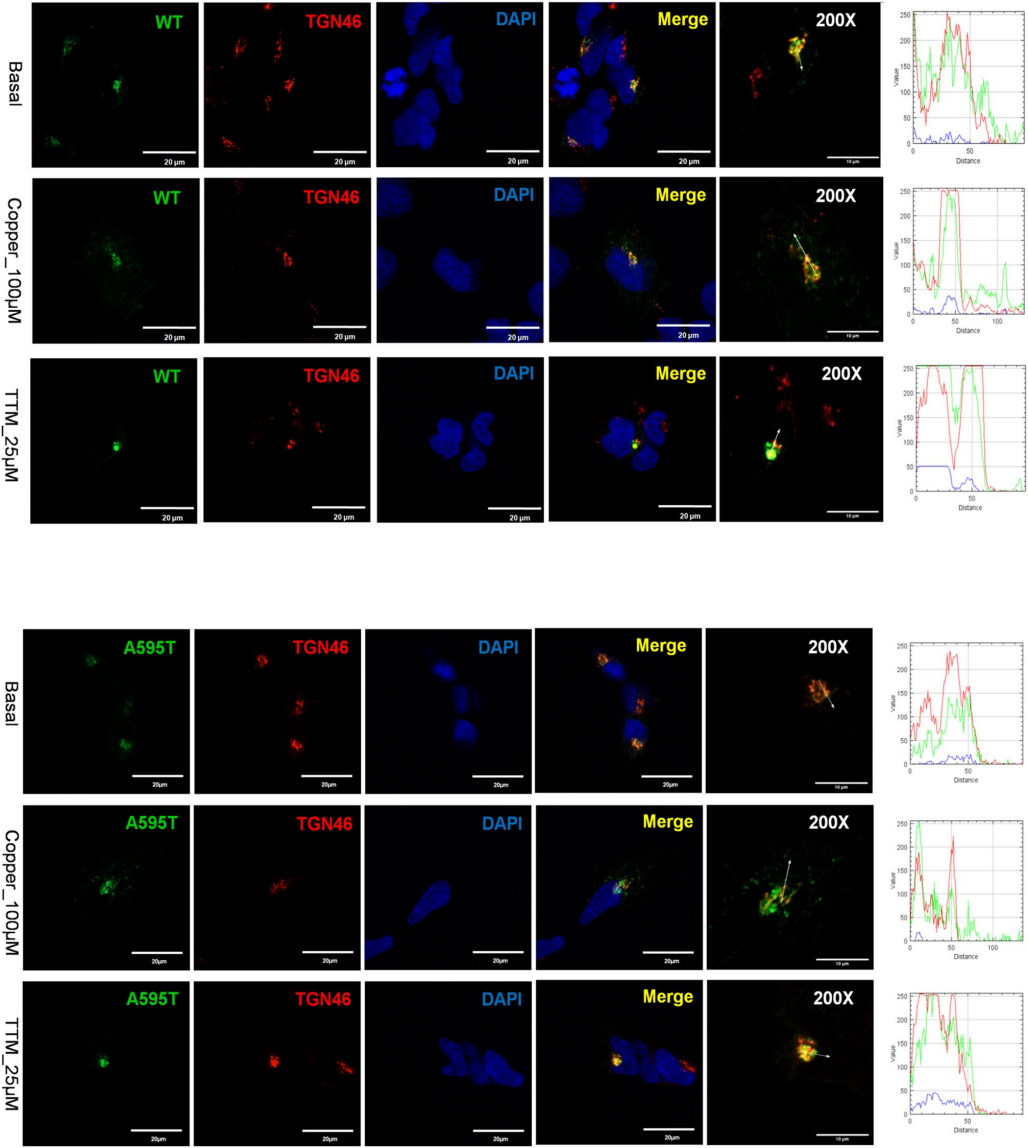
Localization of WT and Ala595Thr mutant (A595T) ATP7B in HEK293 cells in low and high Cu. HEK293 cells expressing GFP-tagged (**A**) WT ATP7B or (**B**) A595T ATP7B mutant (green) were treated for 3 h with either basal medium (top panels), 100 µM CuCl_2_ (middle panels), or first with 100 µM CuCl_2_ and then with 25 µM tetra-thiomolybdate (TTM) (lower panels). TGN marker TGN46 in red, nuclei (DAPI) in blue. Co-localization is indicated in yellow. Green signals are from GFP fluorescence.

To assess the effect of the mutants on ATP7B protein localization and trafficking, they were transfected into HEK293 cells, which have been commonly used for studies of ATP7B trafficking. As expected, WT ATP7B was found in the TGN under basal conditions, trafficked out of the TGN to vesicles when Cu was elevated, and does not show a vesicular pattern when copper is low (Fig. 5a). The A595T mutant behaved very similarly. Under basal conditions, it was targeted to the TGN, as evident by co-localization with the TGN marker (Fig. 5b). In response to Cu elevation, co-localization decreased significantly, and the mutant was found mostly in vesicles, indicative of trafficking. Following Cu depletion using the Cu chelator tetrathiomolybdate (TTM), the A595T mutant moved back to the TGN (Fig. 5b). These data indicate that the A595T mutant retains normal Cu-dependent trafficking under both low and high Cu conditions.

### G1061E and G1101R ATP7B mutants do not have Cu-transport activity and are trapped in the ER

**Figure 6:**
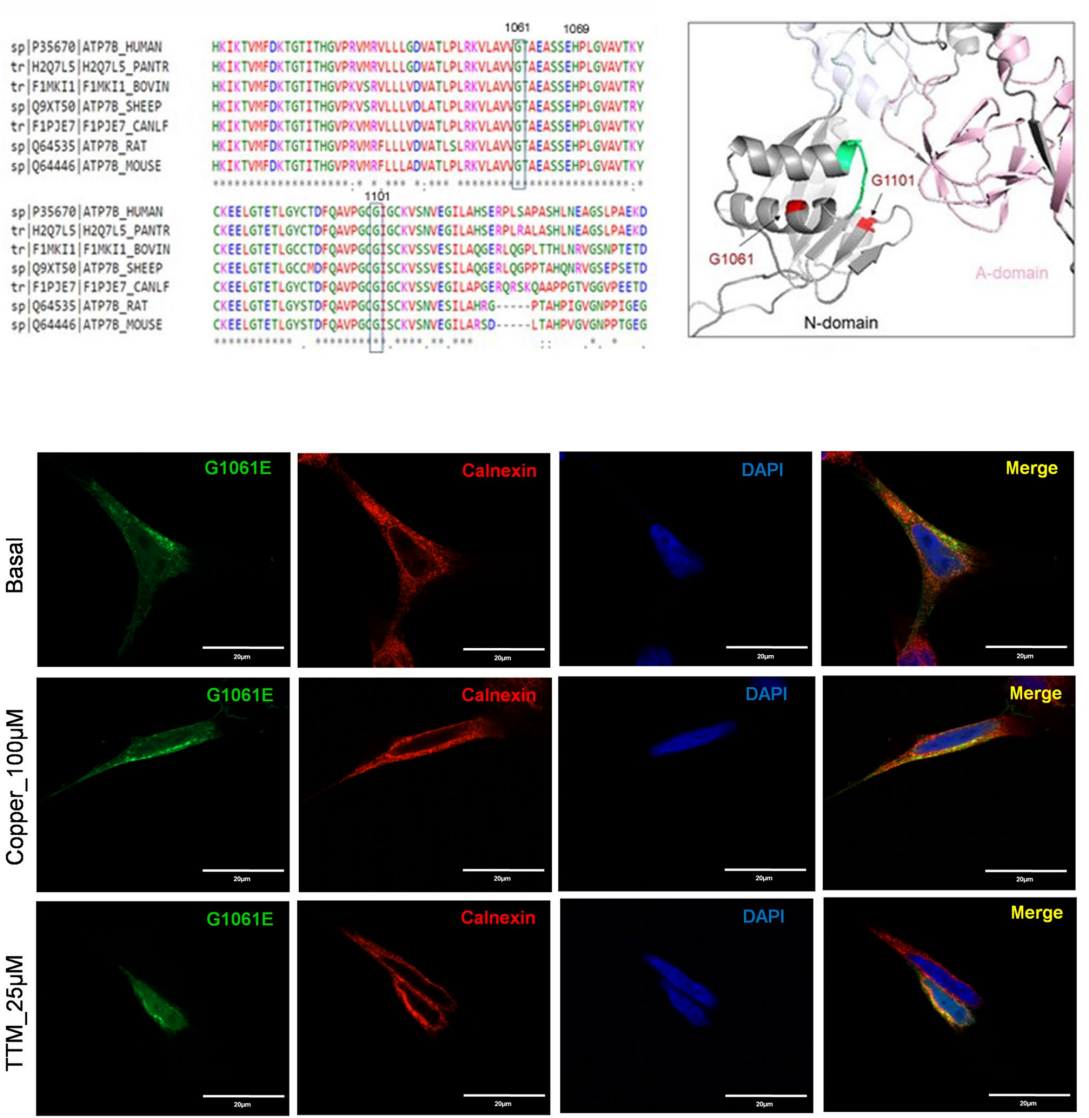
Properties of ATP7B mutants located within the N-domain. (**A**) Multiple sequence alignment showing conservation of Gly1061 (G1061) and Gly1101 (G1101). (**B**) Location of G1061 and G1101 (shown in red) in the N-domain (in grey). The conserved SEHPL motif containing the common H1069Q mutation is in green. The actuator (A)-domain is in pink. (**C**) Localization of the G1061E ATP7B mutant in HEK293 cells under low and high Cu conditions. HEK293 cells were transfected (12 h) with WT or G1061E mutant GFP-ATP7B (green) and treated for 3 h with basal medium (top), medium containing 100 µM of CuCl_2_ (middle), or medium containing 25 µM of tetra-thiomolybdate (TTM) (lower panel).Endoplasmic reticulum (ER) marker, Calnexin, in red and nuclei (DAPI) in blue. Co-localization is indicated in yellow. Green signals are from GFP fluorescence.

The G1061E and G1101R mutations are located in the nucleotide-binding domain (N-domain) of the ATP7B protein (Fig. 1). G1061E was reported as the second most common mutation in eastern Indian WD patients, comprising 11% of mutated alleles observed^17^. The G1061E mutation was also found in the WD patient cohort from south India and Mediterranean regions^18,19^. The residue G1061 is highly conserved (Fig. 6a), and it is located in the α-helix in the immediate vicinity of the invariant SEHPL motif, the location of another common and well-characterized WD-causing mutation, H1069Q (Fig. 6b). G1061 is oriented towards the protein core, and replacement of this small, neutral residue with the bulky, negatively charged Glu is likely to disrupt protein structure. Indeed, analysis of expression and localization of the G1061E mutant in HEK293 cells revealed that it co-localizes completely with the endoplasmic reticulum (ER) marker Calnexin under basal conditions (Fig. 6c). Consistent with protein misfolding and retention in the ER, the protein levels of the mutant ATP7B were reduced compared to WT ATP7B (Fig. 4e, f). Moreover, tyrosinase activation assay in cells expressing this mutation showed no pigment formation (Fig. 3a), suggesting greatly diminished Cu-transport activity. The lack of pigment formation was similar to the result with the D1027A mutant, which is expressed normally (Fig. S1) but is catalytically inactive^20^ and therefore incapable of transporting copper and activate tyrosinase. While retention of ATP7B mutants in the ER in some cases can be overcome by increasing Cu concentration^21^, the G1061E mutant remained trapped in the ER under both high and low Cu conditions.

**Figure 7:**
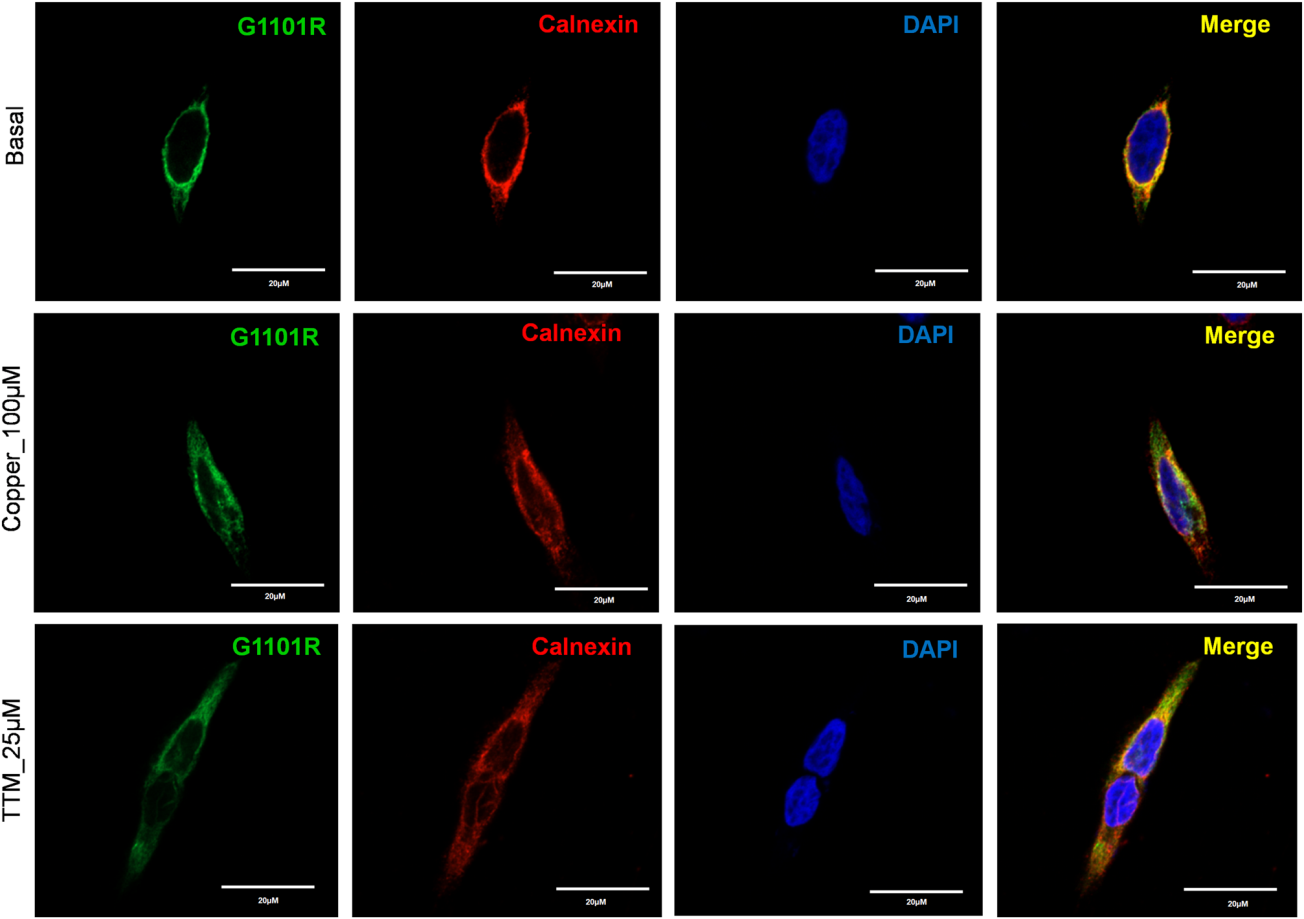
Localization of G1101R ATP7B mutant in HEK293 cells under low and high Cu conditions. HEK293 cells were transfected (12 h) with WT or G1101R mutant ATP7B GFP-ATP7B (green) and treated for 3 h with basal medium (top), medium containing 100 µM of CuCl_2_ (middle), or medium containing 25 µM of tetra-thiomolybdate (TTM) (lower panel).Endoplasmic reticulum (ER) marker, Calnexin, in red and nuclei (DAPI) in blue. Co-localization is indicated in yellow. Green signals are from GFP fluorescence.

The G1101R variant is another prevalent mutation in the southern and western Indian WD patient cohort^18,22^. The Gly residue is invariant and is directly involved in ATP binding within the N-domain^11^. Replacement of small, flexible Gly with bulky, charged Arg likely not only interferes with ATP binding, but also causes structural perturbation within this highly conserved domain (Fig. 6a). Tyrosinase activation assay in cells expressing this mutation showed no pigment formation, indicating the loss of Cu-transport (Fig. 3). Analysis of the G1101R mutant expression in HEK293A cells showed that the mutant protein completely co-localized with the ER marker, Calnexin, independently of Cu concentration (Fig. 7), suggesting that it is retained in the ER. Consistent with these results, the protein levels of the G1101R mutant were reduced compared to WT ATP7B (Fig. 4e, f).

### S1362A ATP7B mutant has reduced Cu-transport activity and lacks Cu-dependent trafficking

**Figure 8:**
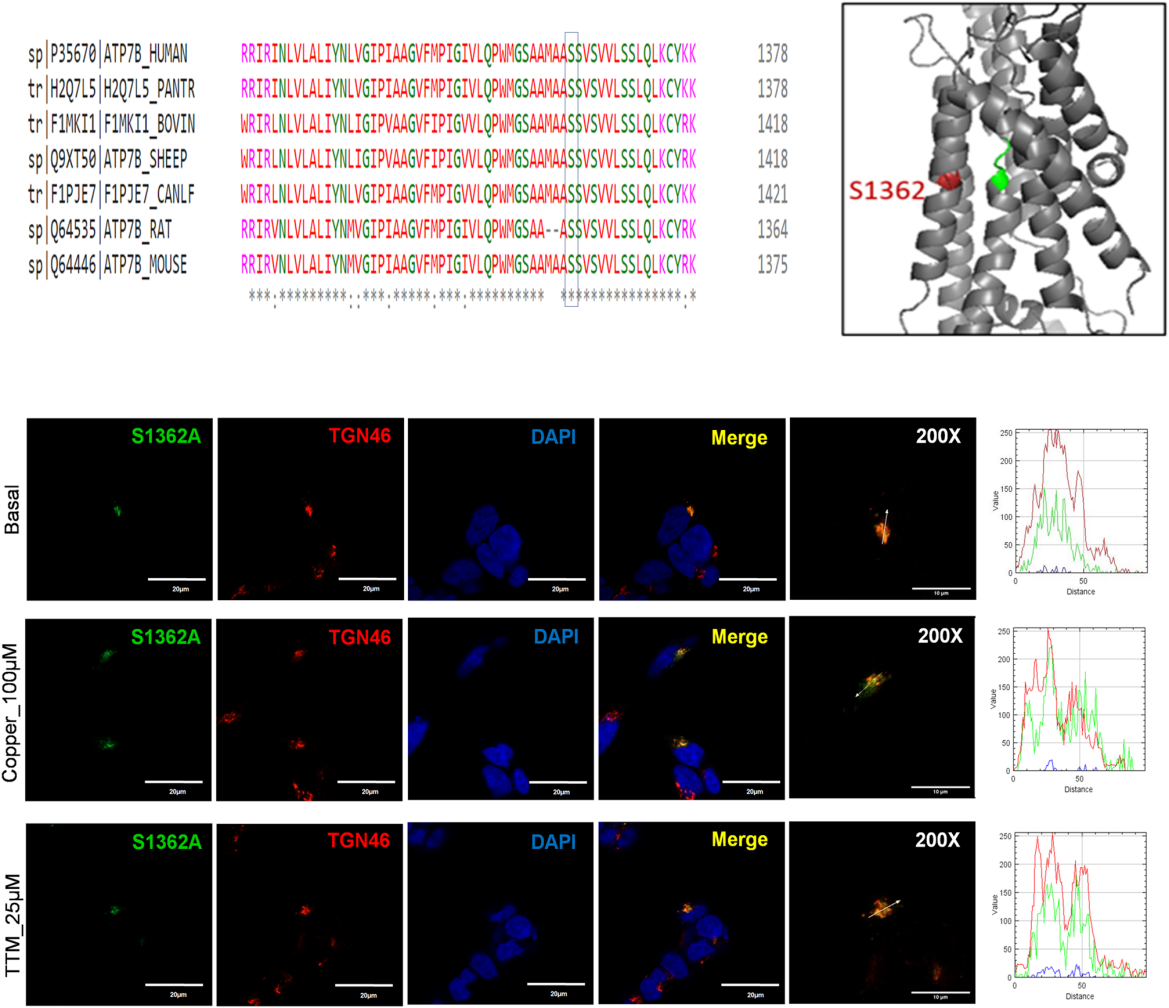
Molecular and cellular characteristics of the S1362A ATP7B mutant. (**A**) Multiple sequence alignment showing conservation of Ser1362. (**B**) Location of Ser1362 (red) in proximity to the invariant CPC motif (green) involved in Cu coordination within the transmembrane domain. (**C**) Localization of S1362A mutant ATP7B in HEK293 cells under low and high Cu conditions. HEK293 cells were transfected (12 h) with WT or S1362A mutant ATP7B GFP-ATP7B (green) and treated for 3 h with basal medium (top), medium containing 100 µM of CuCl_2_ (middle), or medium containing 25 µM of tetra-thiomolybdate (TTM) (lower panel). Trans-golgi network (TGN) marker, TGN46, in red and nuclei (DAPI) in blue. Co-localization is indicated in yellow. Green signals are from GFP fluorescence.

The transmembrane domain (TM) of ATP7B forms the Cu translocation pathway, and the residues within TM4–TM8 are conserved among the species (Fig. 8a). The S1362A mutation is located in TM8 (Fig. 1) and was reported in a 21-year-old Indian male who had high urinary Cu levels and hepatic manifestations of WD^7^ (Table S2). The Ser residue at position 1362 is invariant in ATP7B among various species (Fig. 8a), and it is thought to be directly involved in Cu-binding within the membrane^23,24^. In the homology model of ATP7B^24^, the side chain of S1362 projects into a small cavity in the immediate vicinity of the invariant Cu-binding motif CPC (Fig. 8b). As such, the substitution of Ser with the smaller Ala is unlikely to cause major structural disruptions. At the same time, the loss of the hydrophilic side chain of Ser may negatively affect Cu binding and transport. Analysis of the Cu-transport activity using tyrosinase activation assay revealed significant reduction in the pigment intensity in cells expressing the S1362A mutant compared to WT ATP7B (Fig. 3).The overall pigment intensity was about 11.1% of that of the WT signal (15.11159 ± 29.8 vs. 1.69 ± 1.82, respectively). The level of protein expression was not significantly different from that of the WT ATP7B (Fig. 4).

In HEK293 cells, the S1362A mutant had normal levels of expression and showed complete co-localization with the TGN marker, TGN46, under basal conditions (Fig. 8c). Therefore, the diminished Cu-transport activity observed is likely due to local structural changes within the Cu-transport pathway rather than due to general protein misfolding. Even though the mutant was targeted normally, its sensitivity to elevated Cu was lost. Treatment of cells expressing the S1362A mutant with 100 µM Cu for 3 h showed no trafficking of the mutant from the TGN to vesicles (Fig. 8c). This indicates that the negative effect of this mutation on Cu-transport is compounded by the inability of the mutant ATP7B to move from the TGN to vesicles to facilitate Cu export.

### The S1426I mutant has reduced Cu-transport activity but normal Cu-dependent trafficking

**Figure 9:**
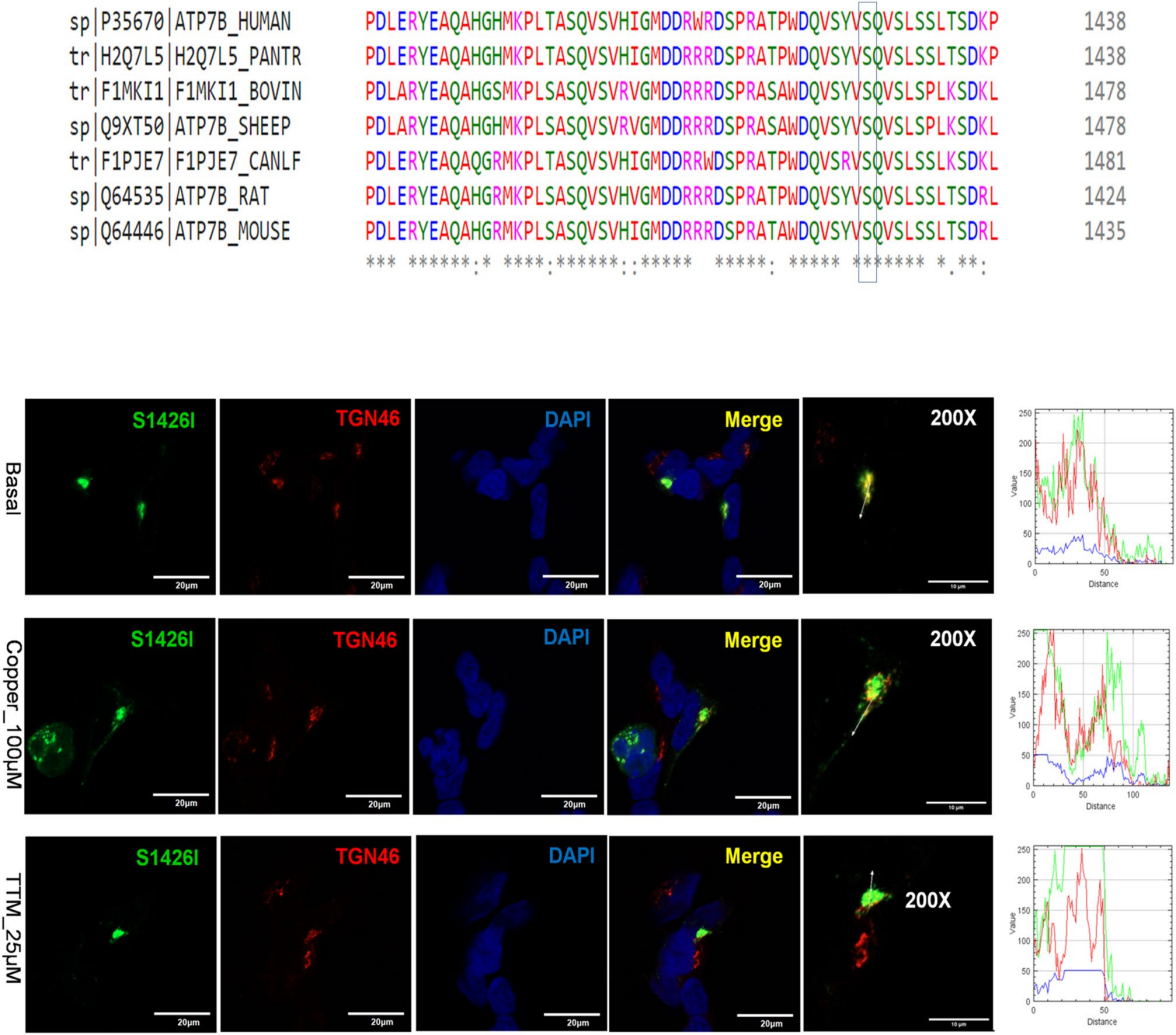
Localization of the S1426I ATP7B mutant in HEK293 cells under low and high Cu conditions. (**A**) Multiple sequence alignment showing conservation of Ser1426Ile. (**B**) HEK293 cells were transfected (12 h) with WT or S1426I mutant ATP7B GFP-ATP7B (green) and treated for 3 h with basal medium (top), medium containing 100 µM of CuCl_2_ (middle), or medium containing 25 µM of tetra-thiomolybdate (TTM) (lower panel). Trans-golgi network (TGN) marker, TGN46, in red and nuclei (DAPI) in blue. Co-localization is indicated in yellow. Green signals are from GFP fluorescence.

The S1426I mutation resides in the C-terminal region of ATP7B (Fig. 1). It was detected in a female Indian WD patient in the compound-heterozygous state, with A1003V on the other allele (Table S2). The C-terminus of ATP7B is predicted to be unstructured, but current data suggest that it plays important regulatory roles by interacting with other proteins and ATP7B domains^25^. Previous studies have shown that partial or complete deletion of the C-terminal domain of ATP7B decreases protein retention in the TGN and impairs retrograde trafficking^26^. Tyrosinase activation assay revealed that the S1426I mutant had reduced but measurable Cu-transport activity (Fig. 3). The mean pigment signal of cells expressing the S1426I mutant decreased by 66.05% compared to WT ATP7B (15.11159 ± 29.8 vs. 5.13 ± 11.52, respectively). In HEK293 cells, the S1426I mutant is targeted to the TGN under basal conditions. Treatment with 100 µM Cu triggers the movement of the S1426I mutant from the TGN to vesicles while under Cu depletion conditions, the mutant shows complete co-localization with the TGN marker, TGN46 (Fig. 9).

### Cu retention in YST cells expressing different ATP7B mutants

**Figure 10:**
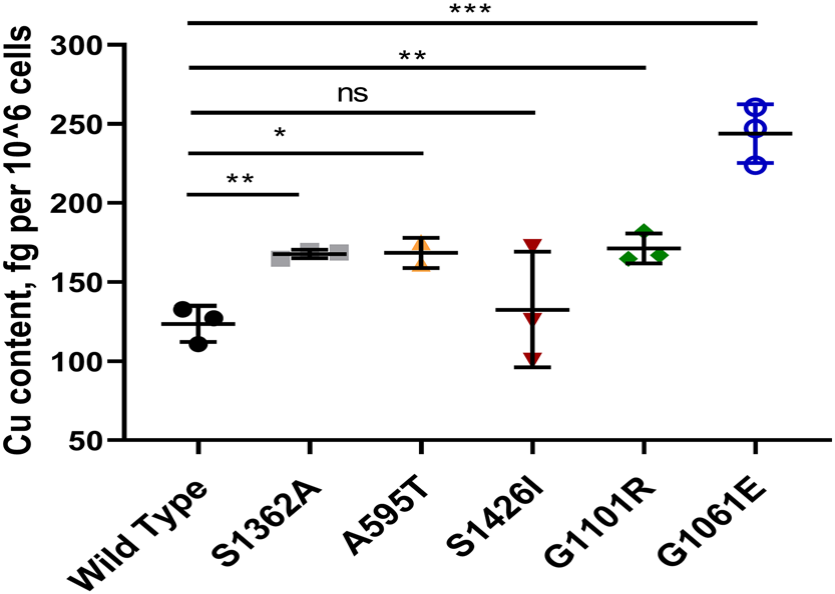
Cu retention in YST cells expressing ATP7B mutants. YST cells were transfected (12 h) with WT or mutant (A595T, S1362A, S1426I, G1061E, and G1101R) ATP7B and treated for 24 h with medium containing 100 µM of CuCl_2_. Cellular Cu content was measured by inductively coupled plasma mass spectrometry (ICP-MS) to evaluate Cu-export. The values were normalized to the YST cell count. All values are reported as means ± SD. Significance was determined by unpaired Student’s *t* test, *p < 0.05; **p < 0.01; ***p < 0.001, *n* = 3.

The above experiments identified three categories of ATP7B mutants: (1) mutants that are mis-localized and inactive, (2) mutants with reduced Cu-transport activity but normal Cu-dependent trafficking, and (3) mutants with reduced Cu-transport activity and impaired trafficking behavior. To determine whether mutants with partial transport and/or trafficking activity affect the total cellular Cu content, YST cells were transfected with either WT ATP7B or individual mutants and then cellular Cu content was measured using ICP-MS. Cells expressing WT ATP7B had the lowest Cu content (123.57 ± 11.35 fg/10^6^ cells), whereas the inactive G1061E and G1101R mutants had the highest Cu contents (243.77 ± 18.53 and 171.11 ± 9.46 fg/10^6^ cells, respectively). Moreover, cells expressing the S1362A mutant had more Cu than cells expressing WT ATP7B (167.55 ± 2.68 vs. 123.57 ± 11.35 fg/10^6^ cells, respectively). This observation is consistent with the finding that the S1362A mutant cannot move out of the TGN towards the plasma membrane and pump excess Cu out of the cell. Cells expressing the S1426I mutant did not show a significant difference in Cu content compared to WT ATP7B, though it did have more variable values (Fig. 10).

Taken together, these data illustrate not only the broad range of consequences that mutations in ATP7B have on the properties of the protein, but also the wide variation of Cu-retention phenotypes in cells expressing the mutations in the homozygous state.

### Different ATP7B mutants interact and influence each other’s behavior

**Figure 11:**
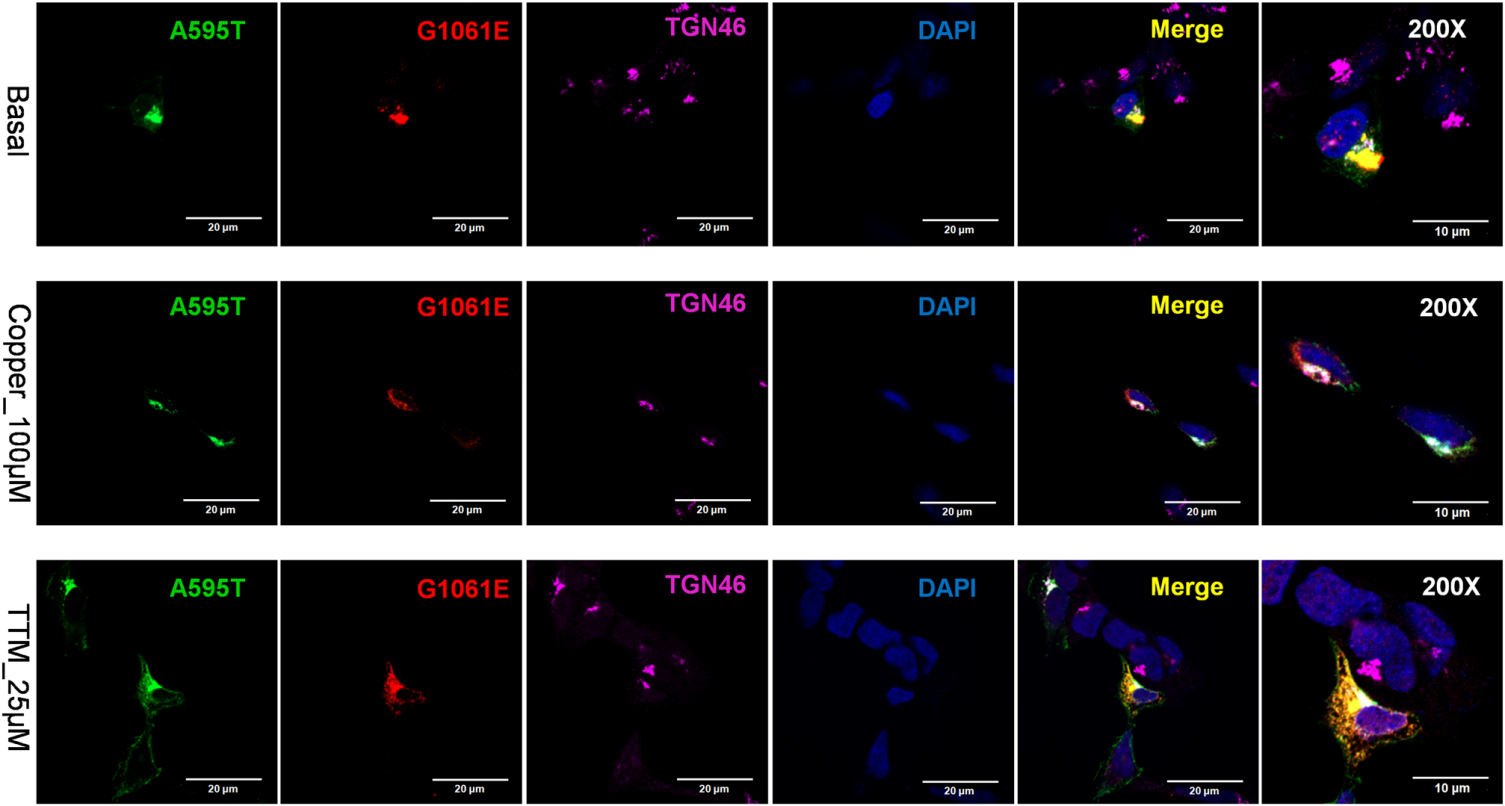
Co-expression of A595T and G1061E ATP7B mutants in HEK293 cells alters their subcellular distribution. HEK293 cells were co-transfected with GFP-ATP7B-A595T (green) and FLAG-ATP7B-G1061E (red) and incubated in basal medium (top panels), medium containing 100 µM CuCl_2_ (middle panels), or medium containing 25 µM of tetra-thiomolybdate (TTM; lower panels). Trans-golgi network (TGN) marker, TGN46, in red and nuclei (DAPI) in blue. Co-localization is indicated in yellow. The rightmost panels show × 200 magnified images. Green signals are from GFP fluorescence.

Many WD patients are compound-heterozygous for ATP7B mutations, i.e. express different ATP7B mutants in same cell. In cells, ATP7B forms a dimer, and heterodimerization of experimental ATP7B variants with different trafficking properties was shown to modify their localization compared to homodimeric forms^15^. This finding suggested that dissimilar WD-causing mutants could influence each other localization and trafficking. To test this hypothesis, we co-expressed the A595T and G1061E mutants in cells. This combination was previously found in a male WD patient who had low plasma ceruloplasmin levels and high urinary Cu levels (Table S2). The G1061E mutant is trapped in the ER when expressed alone (Fig. 7), but is targeted to the TGN under basal conditions when co-expressed with the A595T mutant, with which it is fully co-localized (Fig. 11). In other words, the presence of an active, better folded mutant restores the localization of an inactive and misfolded mutant. However, the dimerization of the active A595T with the inactive G1061E has a negative impact on the Cu-dependent trafficking of ATP7B from the TGN. The A595T mutant, which when expressed alone, traffics from the TGN in response to Cu (Fig. 5), shows no trafficking in response to Cu when co-expressed with the G1061E mutant. Given that the loss of trafficking often reflects the loss of Cu-transport, these results suggest that the A595T mutant forms a non-functional complex with the G1061E mutant. Lastly, under low Cu conditions, only a small fraction of the A595T mutant was found to co-localize with the TGN marker. The majority of A595T co-localized with the G1061E mutant in the pattern consistent with their retention in the ER. Thus, while dimerization occurs in the ER in the absence of Cu, the proper folding of the dimer and its exit from the ER is at least partially Cu-dependent.

## Discussion

WD is a monogenetic disorder with a remarkable variety of both mutations and manifestations of disease. Previous studies have identified ATP7B mutations in different domains of the protein that had a negative impact on ATP7B stability^13^, loss of Cu-transport activity^14^, ER retention^15^, and inability to traffic from the TGN to vesicles in response to Cu elevation^16,17^. However, establishing clear genotype–phenotype correlations in WD has proven difficult. The majority of disease-associated mutations in ATP7B are missense mutations, and WD patients are commonly compound-heterozygous. In this study, we characterized the cellular properties of mutations identified among Indian WD patients and demonstrated the importance of studying mutations in combinations observed in compound-heterozygous WD patients. The properties of the individual mutations are summarized in Table 1.

**Table 1:** Properties of the individual ATP7B mutants characterized in this study.

Mutation	Domain	Cu-transport	Eumelanin signal (area × intensity)	Subcellular localization	Cu-dependent trafficking	Intracellular Cu compared to WT
Ala595Thr	MBD6	Yes	7.91 ± 23.04	Golgi and vesicles	Yes	High
Gly1061Glu	N- domain	No	NA	ER retained	No	High
Gly1101Arg	N domain	No	NA	ER retained	No	High
Ser1362Ala	TM-8	Yes	1.69 ± 1.82	Golgi retained	No	High
Ser1426Ile	C-terminal	Yes	5.13 ± 11.52	Golgi and vesicles	Yes	No difference

*MBD* metal binding domain, *Cu* copper, *ER* endoplasmic reticulum, *WT* wild type.

The A595T, S1362A, and S1426I mutations are novel genetic changes, whereas the G1061E and G1101R mutations were previously identified in the WD patient cohort^7^. These five ATP7B mutations are located in structurally and functionally different domains of the protein and are distributed throughout the entire ATP7B structure, including the N-terminus, the TM domain, the ATP-binding domain, and the C-terminus. We identified a broad spectrum of consequences on ATP7B activity and cellular behavior, in agreement with previous analysis of other WD-causing mutations^27^.

The N-terminal domain of ATP7B has 6 metal binding domains (MBD 1–6) that have similar structures but different functions^28^. MBD1, MBD2, and MBD3 form a transient complex that receives Cu from Atox1^29^. MBD5 and MBD6 are situated close to the TM domains of ATP7B^30^, and at least one of these domains must be present to sustain Cu-transport activity^31^. Consistent with this, the A595T mutation, within MBD6, results in a significant reduction in Cu-transport activity of the protein and a decrease in its ability to remove Cu from cells. However, this missense mutation did not lead to gross misfolding or mis-localization, nor did it disrupt Cu-transport completely, as evidenced from the fact that it was able to traffic from the TGN to vesicles in response to Cu elevation. These properties are similar to those of the previously characterized G591D and R616Q mutants, which are also located in MBD6^32^. Taken together, these data suggest that the structural integrity of A595 is important for Cu-transport but not crucial for Cu-dependent trafficking. The impact of this mutation on trafficking kinetics, if any, remains to be determined. Notably, the fact that the A595T mutation results in only a 50% decrease in Cu-transport activity suggests that this mutation alone may not cause the WD phenotype but perhaps would in combination with more deleterious mutations.

In the N-domain of ATP7B, the residues H10169, G1099, G1101, I1102, G1149, and N1150 are indispensable for the binding of ATP^33^. Mutation of some of these residues or their neighboring residues are associated with WD^11^. The H1069Q substitution is a common WD-causing mutation found in Caucasian populations^3,34^. The G1061E and G1101R mutations have been identified as prevalent missense mutations among Indian WD patients^35^. Mutations at these two positions may directly impede ATP binding and disrupt protein folding, as both have the small Gly residue replaced with bulkier, charged residues. We show that these two mutations produce misfolded ATP7B and completely block Cu-transport to the secretory pathway. Previous studies have found that mutations within the N-domain often disrupt protein folding and stability^36^. Unlike the G875R variant of ATP7B that can be rescued from ER retention by the addition of Cu^21^, the G1061E and G1101R mutants remain in the ER even under high Cu conditions. Taken together our data suggest that zero copper transport activity of G1101R and G1061E observed in a tyrosinase could be a combination of their compromised function, retention in the ER and lower level of expression. Previous studies have identified some novel therapeutics and molecular targets in the signaling pathway that seems to be promising in rescuing ER retained and misfolded ATP7B mutations^37,38^. It would be interesting to observe whether this same strategy can be applied to correct these two ER retained ATP7B mutations like G1101R and G1061E.

Although both the G1061E and G1101R mutants have similar properties in cells, the reported clinical phenotypes of WD patients with these mutations are very different. The p.G1061E homozygotes had predominantly neuropsychiatric presentations with an age of onset of 9–16 years, whereas the p.G1101R homozygotes had liver involvement and an age of onset of 5–7 years^7^ (Table S2). These results caution against establishing direct links between molecular and clinical phenotypes and support emerging evidence suggesting that other modifying factors play a role in WD.

The trafficking of ATP7B is a complex process, and the factors regulating the exit of ATP7B from the TGN are still not fully understood. Previous reports have demonstrated that TM1 and TM2 play a role in the Cu-dependent trafficking of ATP7B to vesicles^39^. It has also been suggested that Cu-transport and the ability to traffic are linked^13^. For instance, while mutation of D1027 has no negative impact on ATP7B structure, mutation of this residue renders the protein catalytically inactive and blocks its exit from the TGN. Previous studies have found that the S1362F-fs mutation impairs Cu-transport and phosphorylation activity^27^. S1362A is a novel mutation in TM8 of ATP7B found in an Indian WD patient^7^. Within the molecular model of ATP7B, this residue is located in the immediate vicinity of the crucial Cu-binding CPC motif and M1359 residue of TM8 (Fig. 8b). This finding suggests that structural changes caused by the mutation may impact Cu binding and translocation within the domain, as well as the ability of the protein to leave the TGN. Consistent with these predictions, the tyrosinase activation assay shows a significantly lower activity for this variant compared to WT ATP7B. The mutant also shows complete TGN retention, even under high Cu conditions. Similar to these results, S653Y in TM1 and G943S in TM5 were shown to result in ATP7B protein with some amount of Cu-transport activity, though the proteins failed to exit the TGN under high Cu conditions^39,40^. Taken together, these data suggest that the TM portion of ATP7B plays an important role in its exit from the TGN, possibly through interaction with specific lipids or other molecules.

The unstructured C-terminal region of ATP7B contains conserved signal residues (e.g. tri-leucine^1454^LLL^1456^) important for vesicular trafficking of the protein^41^. Numerous Ser/Thr residues in this domain were found to be phosphorylated^11^. Partial or complete deletion of the C-terminus destabilizes the ATP7B protein and impairs retrograde trafficking. The L1373R mutation in the C-terminus has been associated with reduced Cu-transport and protein expression, as well as loss of TGN retention under low Cu conditions^26^. In contrast, the S1426I mutant had normal protein expression and trafficked normally in response to Cu stimulation. Even though its Cu-transport activity was reduced, the ability of this variant to remove Cu from cells was not significantly compromised, indicating that this mutation is likely mild. This conclusion is consistent with the compound-heterozygous status of the patient from which this mutation was identified.

Many WD patients are compound-heterozygous, i.e., have different ATP7B mutations on two different alleles. Mukherjee et al. identified a WD patient with the A595T and G1061E mutations in a compound-heterozygous state^7^. By characterizing these mutations both separately and together, we showed that these mutants interact, and that this interaction in turn impacted their cellular properties. Alone, the catalytically inactive G1061E is retained in the ER, whereas the partially active A595T is targeted to the TGN and traffics in response to high Cu. The co-expression of these mutants to mimic the compound-heterozygous state generated an ATP7B complex with distinct cellular properties. Under low Cu conditions, only a small fraction of the A595T mutant was found to co-localize with the TGN marker, and the majority was found to co-localize with the G1061E mutant in the ER. No vesicular movement of the mutants was observed following Cu stimulation. The low levels of plasma ceruloplasmin (Table S2) observed in the WD patient containing these mutations could be explained by this cellular phenotype, however direct measurements of copper-transport activities of heterodimer (using tyrosinase assay or copper retention) will be necessary to fully understand consequences of compound heterozygosity on ATP7B properties.

Taken together, our findings suggest that future genotype–phenotype correlation studies should rely, when appropriate, on characterization of ATP7B hetero-dimers rather than individual ATP7B mutants. Furthermore, sometimes positive influence of mutants (such as rescue of ATP7B localization by a better folded mutant upon dimerization—as we observed A595T and G1061E mutations) can modify the property of more deleterious mutant producing milder phenotype. In our view, going forward the best approach for studies of ATP7B mutants would be to use the WD patients-derived cells.

## Supplementary information

Supplementary Information.
