# Supplementary material for: Analysis of Wilson disease mutations revealed that interactions between different ATP7B mutants modify their properties
Source: Sci Rep. 2020 Aug 10;10:13487. doi: 10.1038/s41598-020-70366-7 (PMC7418023; doi:10.1038/s41598-020-70366-7)
Supplement: Supplementary file 1 — Supplementary Information. [file 41598_2020_70366_MOESM1_ESM.docx]

**ANALYSIS OF WILSON DISEASE MUTATIONS REVEALED THAT INTERACTIONS BETWEEN DIFFERENTATP7B MUTANTS MODIFY THEIR PROPERTIES**

**Shubhrajit Roy^1,4*,^ Courtney J. McCann^1^, Martina Ralle^2^, Kunal Ray^3^, Jharna Ray^4^, Svetlana Lutsenko^1ζ*^^[[1]](#footnote-1)^, and Samuel Jayakanthan^1ζ^**

^1^ Department of Physiology, Johns Hopkins Medical Institute, Baltimore MD, USA; ^2^Oregon Health & Science University, Portland OR, USA;^3^ATGC Diagnostics Private Ltd, Kolkata, India;^4^ S. N. Pradhan Centre for Neurosciences, University of Calcutta, Kolkata, India.

*Corresponding Authors:

Shubhrajit Roy ([shubhrajitroy123@gmail.com](mailto:shubhrajitroy123@gmail.com))

Svetlana Lutsenko ([lutsenko@jhmi.edu](mailto:lutsenko@jhmi.edu))

*To Whom Correspondence should be addressed:* [shubhrajitroy123@gmail.com](mailto:shubhrajitroy123@gmail.com)

**SUPPLEMENT**

**Supplementary Table 1: Primer sequences for Site Directed Mutagenesis of ATP7B.**

| Sl.No | Mutation | Forward Primer (5’-3’) | Reverse Primer (5’-3’) |
| --- | --- | --- | --- |
| 1 | G1101R | CCAGGCAGTGCCAGGCTGTaGAATTGGGTGCAAAGTCAGC | GCTGACTTTGCACCCAATTCtACAGCCTGGCACTGCCTGG |
| 2 | G1061E | GGAAGGTTCTGGCTGTGGTGGaGACTGCGGAGGCCAGCAGTG | CACTGCTGGCCTCCGCAGTCtCCACCACAGCCAGAACCTTCC |
| 3 | D1027A | AAAGACTGTGATGTTTGcCAAGACTGGCACCATT | AATGGTGCCAGTCTTGgCAAACATCACAGTCTTT |
| 4 | S1362A | GGGCTCAGCGGCCATGGCAGCCgCCTCTGTGTCTGTGGTGC | GCACCACAGACACAGAGGcGGCTGCCATGGCCGCTGAGCCC |
| 5 | A595T | CGAGGACAAATGGCATCACTTATaCCTCCGTTGCCCTTGCCACC | GGTGGCAAGGGCAACGGAGGtATAAGTGATGCCATTTGTCCTCG |
| 6 | S1426I | GGGACCAGGTCAGCTATGTCAtCCAGGTGTCGCTGTCCTCCC | GGGAGGACAGCGACACCTGGaTGACATAGCTGACCTGGTCCC |

*ATP7B*, Copper transporting ATPase 2;G1101R, Gly1101Arg;G1061E, Gly1061Glu;D1027A, Asp1027Ala;S1362A, Ser1362Ala;A595T, Ala595Thr; S1426I,Ser1426Ile

**Supplementary Table 2: Clinical details and ATP7B mutations of the Indian Wilson disease**

| **Patient** | **ATP7B mutations** | **Sex** | **Age at Onset** | **KF ring** | **Cp**  **(mg/dL)** | **24 hour Urinary Copper (µg/24hrs)** | **Phenotype** |
| --- | --- | --- | --- | --- | --- | --- | --- |
| WD1 | Ala595Thr/  Gly1061Glu | Male | 15 years | Present | 3.15 | 132.81 | Wing-beating tremor, dystonia, rigidity, postural instability |
| WD2 | Ser1362Ala/  Ser1362Ala | Male | 21  Years | Present | NA | 650 | Elevated Liver enzymes (SGPT, SGOT), splenomegaly, Ascites-Liver |
| WD3 | Gly1061Glu/  Gly1061Glu | Female | 16  Years | Present | 4 | NA | Tremor, dystonia, rigidity |
| WD4 | Gly1061Glu/  Gly1061Glu | Male | 9  Years | Present | NA | 196 | Tremor,  rigidity, dystonia, elevated liver enzymes (SGPT,SGOT) |
| WD5 | Gly1061Glu/  Gly1061Glu | Male | 12 years | Present | 6 | NA | Dystonia, rigidity |
| WD6 | Gly1101Arg/  Gly1101Arg | Male | 5 years 6 months | Absent | 10 | 290 | Mild cirrhosis of Liver |
| WD7 | Gly1101Arg/  Gly1101Arg | Female | 7 years | Absent | 4 | 114 | Mild hepatic symptoms |
| WD8 | Gly1101Arg/  Gly1101Arg | Female | 6 years | Absent | 18 | 87 | Mild hepatic symptoms |
| WD10 | Ser1426Ile/ Ala1003Val | Female | 9 years | NA | NA | NA | NA |

ATP7B, Copper transporting ATPase 2; KF ring, Kayser-Fleischer Ring; Cp, Ceruloplasmin; SGPT, serum glutamate-pyruvate transaminase; SGOT, serum glutamate-oxalate transaminase; NA, data not available.

KF ring determined using slit lamp test

**Supplementary Table 3: Structural details of the amino acid residues**

| Mutation | Van der Waals Volume^f^ (A^3^) | | Accessible surface area (A^2^) | |
| --- | --- | --- | --- | --- |
| Ala595Thr | Wild Type | Mutant | Wild Type | Mutant |
|  | Alanine: 67 | Threonine: 93 | Alanine: 67 | Threonine: 102 |
| Gly1061Glu | Glycine: 48 | Glutamic acid: 109 | Glycine: NA | Glutamic acid: 138 |
| Gly1101Arg | Glycine: 48 | Arginine: 148 | Glycine: NA | Arginine: 196 |
| Ser1362Ala | Serine: 73 | Alanine: 67 | Serine: 80 | Alanine: 67 |
| Ser1426Ile | Serine: 73 | Isoleucine: 124 | Serine: 80 | Isoleucine: 140 |

fData from Darby and Creighton (1993)

NA; Not applicable

**Supplementary Figure**

**
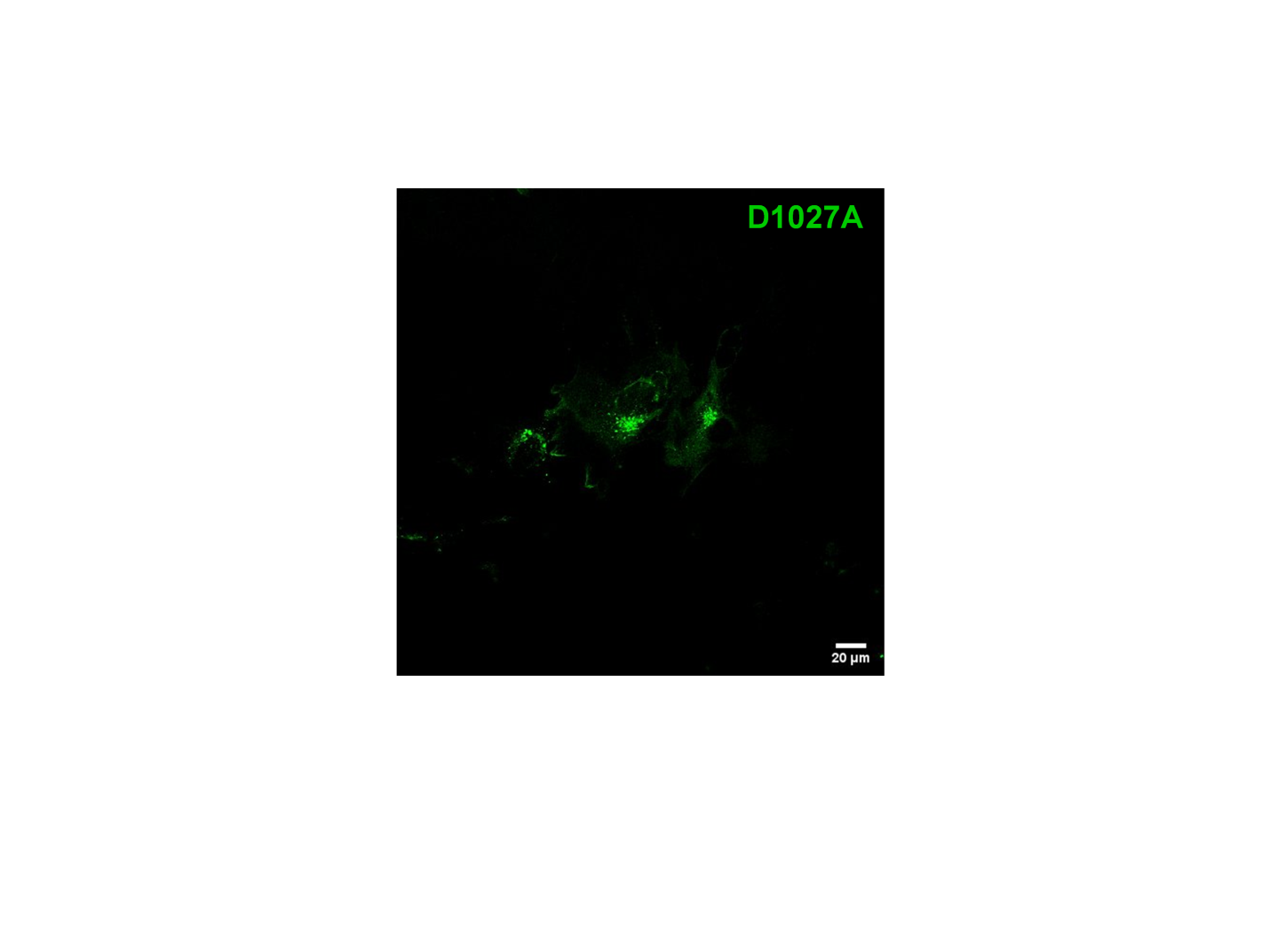
**

Supplementary Figure S1: The expression of GFP tagged ATP7B mutant D1027A expressed in YST cells visualized using GFP fluorescence. Images were captured using confocal microscope (Zeiss) using a 40x oil lens.

Supplementary Figure S2:

1. Original full length blot (.TIF image) for Figure 4C. The boxes indicate the area shown in the figure.
2. Full length blot image with pre-stained protein marker.
3. Full length membrane showing only pre-stained protein marker.

Supplementary Figure S3: Original full length blot (.TIF image) for Figure 4E. The boxes indicate the area shown in the figure.

Supplementary Figure S4: Pearson correlation coefficient of co-localization between ATP7B (green) and TGN46 (red) at different copper conditions [Basal, 100µM copper (high) and 25μTTM (low)]. A. Pearson correlation coefficient of co-localization for Wild type ATP7B. B. Pearson correlation coefficient of co-localization for A595T. C. Pearson correlation coefficient of co-localization for G1061E. D. Pearson correlation coefficient of co-localization for G1101R E. Pearson correlation coefficient of co-localization for S1362A F. Pearson correlation coefficient of co-localization for S1426I mutation.

The Pearson coefficient of colocalization was calculated using JaCoP plugin of ImageJ software. n =3

1. ^ζ -equal contribution^ [↑](#footnote-ref-1)
